# Effects of Minority Status on Sensory Outcomes, Cognition, and Depression

**DOI:** 10.1002/ohn.70416

**Published:** 2026-08-26

**Authors:** Ivette Cejas, Jennifer Coto, Aria Nawab, Alberto Cruz, Sandra Velandia, Pawan Pathagamage, Molly Smeal, Rosie C. Cid, David Loewenstein, Xue Z. Liu

**Affiliations:** 1 Department of Otolaryngology, University of Miami Miller School of Medicine, Miami, Florida, USA; 2 Department of Pediatrics, University of Miami Miller School of Medicine, Miami, Florida, USA; 3 Department of Public Health Sciences, University of Miami Miller School of Medicine, Miami, Florida, USA; 4 Hearts for Hearing, Oklahoma City, Oklahoma, USA; 5 Department of Psychiatry and Behavioral Sciences, Center for Cognitive Neuroscience and Aging, University of Miami Miller School of Medicine, Miami, Florida, USA

**Keywords:** aging, cognition, mental health, minority, sensory function

## Abstract

**Objective.:**

To evaluate the association between minority status and sensory function, sleep quality, depressive symptoms, and cognitive performance in an aging population.

**Study Design.:**

Prospective, cross-sectional study.

**Setting.:**

Participants were recruited from the Department of Otolaryngology and the Center for Cognitive Neuroscience and Aging at a large academic medical center.

**Methods.:**

Adults aged 50 to 90 years underwent audiologic, visual, olfactory, and cognitive testing. Olfaction was assessed using the University of Pennsylvania Smell Identification Test. Depressive symptoms and sleep quality were measured using the Geriatric Depression Scale and Pittsburgh Sleep Quality Index, respectively. Cognitive function was evaluated using the Mini-Mental State Examination–2 (brief and standard versions). Participants were stratified by minority status. Group comparisons and multiple linear regression analyses were performed to identify independent predictors of cognitive performance.

**Results.:**

A total of 164 participants were included. Minority participants were younger but demonstrated poorer olfactory performance, memory recall, and Mini-Mental State Examination–2 scores. Minority individuals also reported more depressive symptoms and longer sleep latency. Hearing thresholds were also worse among non-minority participants. Multivariable regression identified minority status, olfactory function, and depressive symptoms as independent predictors of cognitive performance, although overall model explanatory power was modest.

**Conclusion.:**

Minority status, depressive symptoms, and olfactory dysfunction were independently associated with lower cognitive performance despite younger age. These findings highlight potential disparities in sensory and cognitive health and support incorporation of olfactory and mood assessment in geriatric otolaryngologic evaluation.

Alzheimer’s disease (AD) affects more than 5.8 million individuals in the United States, with prevalence projected to reach 13.8 million by 2050.^1^ Early identification of modifiable or prodromal risk factors is critical. Sensory impairments, particularly hearing and olfaction, along with sleep disturbance and depression, have emerged as potential contributors to cognitive decline, though these relationships are complex and potentially bidirectional.^2^

Hearing loss is an established risk factor for dementia.^3,4^ However, pure-tone audiometry may not capture speech-in-noise deficits or central auditory dysfunction, potentially underestimating associations with cognitive decline.^5^ Hearing impairment has also been associated with reduced cognitive performance in Hispanic and Latino populations.^6^

Olfactory dysfunction is among the earliest clinical markers of AD and may precede overt cognitive symptoms by years.^7,8^ Because anosmia is frequently attributed to sinonasal disease, careful evaluation is essential to distinguish peripheral pathology from possible prodromal neurodegenerative change.^9^ This distinction is particularly relevant in otolaryngology practices, where early recognition may facilitate timely referral and intervention. Visual impairment and depressive symptoms have also been associated with cognitive decline and dementia.^10–15^

Despite growing evidence connecting sensory dysfunction and mood disorders to cognitive decline, minority populations remain underrepresented in AD research. African American and Hispanic individuals bear a disproportionate burden of AD yet are less frequently included in research cohorts, limiting generalizability.^16^

In this study, we examined whether sensory function (hearing and olfaction), sleep disturbance, and depressive symptoms were associated with cognitive performance in two demographically distinct cohorts with increased representation of Hispanic participants. We hypothesized that minority status and sensory measures would be associated with cognitive performance and examined whether olfactory function, hearing thresholds, sleep disturbance, and depressive symptoms independently predicted cognitive scores. By integrating a clinically derived otolaryngology cohort with an aging center cohort, this study aims to clarify the relative contributions of sensory and behavioral factors to cognitive performance in a more diverse population.

## Methods

### Study Design and Participants

This prospective, cross-sectional study was conducted to evaluate associations among cognitive performance, sensory function, depressive symptoms, sleep quality, and demographic factors. The study was approved by the University of Miami Institutional Review Board (IRB #20210741).

Participants included 105 adults recruited from the University of Miami Department of Otolaryngology Ear Institute and 59 older adult research participants from the University of Miami Center for Cognitive Neuroscience and Aging (CNSA). Eligible participants were 50 to 90 years of age, fluent in English and/or Spanish, and capable of providing informed consent. Exclusion criteria included severe cognitive impairment, history of stroke or traumatic brain injury, diagnosis of Parkinson’s disease, or inability to communicate in English or Spanish. Demographic and clinical information, including age, race, ethnicity, sex, education, income, employment, marital status, general health, COVID-19 history, and audiologic/vestibular history were collected via questionnaires. Participants were screened, provided written informed consent, and completed all procedures during a single study visit.

### Cognitive Assessment

Cognitive function was assessed using the Mini-Mental State Examination–2 (MMSE-2), including both the Brief Version (MMSE-2:BV) and Standard Version (MMSE-2:SV). Participants were required to score >24 to be included, consistent with established thresholds indicating absence of severe cognitive impairment.

### Audiologic Assessment

Audiologic testing included air-conduction pure-tone thresholds (125–8000 Hz) and bone-conduction thresholds (500–4000 Hz). Pure-tone averages (PTAs) were calculated for standard low/mid frequencies (500, 1000, 2000 Hz) and high frequencies (2000, 4000, 8000 Hz) for each ear.

### Olfactory Assessment

Olfactory function was assessed using the 40-item University of Pennsylvania Smell Identification Test (UPSIT), a forced-choice scratch-and-sniff test. Total UPSIT score was defined as the number of correctly identified odorants (range 0–40), with lower scores indicating poorer olfactory function.

### Visual Acuity Assessment

Distance visual acuity was assessed using the Titmus V2 Vision Screener (Titmus Optical Inc.). Participants were tested with habitual corrective lenses when applicable. Each eye was evaluated individually and then binocularly.

### Self-Report Measures

Depressive symptoms were assessed using the 15-item Geriatric Depression Scale (GDS). Participants scoring >10 were referred for further clinical evaluation and provided community resources.

Sleep quality was assessed using the Pittsburgh Sleep Quality Index (PSQI), which evaluates sleep over the prior month across seven domains: subjective sleep quality, sleep latency, sleep duration, habitual sleep efficiency, sleep disturbances, use of sleep medication, and daytime dysfunction. A global PSQI score was calculated.

### Statistical Analysis

Descriptive statistics were calculated for the full sample and stratified by minority status (minority vs nonminority), which was determined by race and ethnicity. Individuals who self-identified as Hispanic, Black or African American, Asian, or bi-racial were classified as minority. Individuals who self-identified as White/Caucasian were included in the nonminority group. Continuous variables were compared using two-sample *t*-tests or Wilcoxon rank-sum tests, as appropriate. Categorical variables were compared using chi-square tests. Minority participants were combined into a single group to improve statistical power given small subgroup sample sizes. Therefore, the primary results focus on comparisons between White non-Hispanic and minority participants, while exploratory subgroup analyses examining racial and ethnic differences are reported separately.

All regression models were conducted using the combined minority group classification. Multiple linear regression models were constructed to evaluate predictors of MMSE-2:BV and MMSE-2:SV total scores. Stepwise model selection was performed, and models were compared using Akaike’s Information Criterion (AIC). Statistical significance was defined as *P* < .05. Analyses were conducted using R statistical software (version 4.3.2).

## Results

### Participant Characteristics

A total of 164 participants were enrolled (mean age 67.6 years, SD = 9.0). The majority were female (57.1%), English-speaking (77.4%), and insured through Medicare (58.3%). Most participants identified as non-Hispanic (63.8%) and White (51.1%). Educational attainment ranged from less than high school (8%) to doctoral degree (8.6%), with 20.6% holding a bachelor’s degree and 14.2% a master’s degree. Participants were most commonly single (30.3%) or married (34.3%). The study sample included participants across four racial/ethnic groups: Black/African American (n = 49), Hispanic/Latino (n = 62), White non-Hispanic (n = 48), and Other non-Hispanic (n = 4). Participants were recruited across two clinics: UM Ear Institute (n = 105, 59%) and CNSA (n = 73, 41%). Minority representation was similar across recruitment sources, with 73 of 105 participants (70.2%) in the UM Ear Institute cohort and 42 of 59 participants (71.2%) in the CNSA cohort classified as minority.

Among participants recruited from the CNSA, cognitive classifications included amnestic mild cognitive impairment (MCI), multiple domain (25%); amnestic MCI, single domain (8.3%); nonamnestic MCI (3.3%); preclinical MCI (15%); and cognitively impaired, not meeting MCI criteria (8.3%). Consistent with the primary analytic approach, Table 1 reports demographic and clinical characteristics by the collapsed minority status grouping used in subsequent regression analyses. Table 2 presents exploratory subgroup comparisons by race and ethnicity, including Black/African American, Hispanic/Latino, White non-Hispanic, and Other non-Hispanic participants.

### Comparisons by Race/Ethnicity & Minority Status

Overall, the nonminority subgroup was significantly older than the minority subgroup (median 75 years [IQR 67.5–79] vs 65 years [IQR 59–71]; *U* = 3947.5, *P* < .001). Exploratory analyses also revealed that White non-Hispanic participants were significantly older than both Black/African American participants (72.1 ± 9.5 vs 62.9 ± 6.1 years, *P* < .001) and Hispanic/Latino participants (67.1 ± 8.9 years, *P* = .018; Kruskal-Wallis *P* < .001).

Depressive symptoms (GDS total score) was higher in the minority subgroup (median 2 [IQR 1–3]) compared with the non-minority subgroup (median 1 [IQR 0–2]; *U* = 1721.5, *P* < .001). Specifically, Black/African American (3.11 ± 3.09) and Hispanic/Latino participants (2.36 ± 2.51) reported higher GDS scores than White non-Hispanic participants (1.29 ± 2.04; *P* = .003 and .009, respectively).

For sleep measures, only PSQI Component 2 (sleep latency) differed significantly between groups, with higher scores in the minority subgroup (median 2 [IQR 1–2]) compared with the nonminority subgroup (median 0 [IQR 0–1]; *U* = 1406.5, *P* < .001). Global PSQI scores did not differ significantly between race (*P* = .057) or minority status groups (median 7 [IQR 4–11] vs 7 [IQR 4–9]; *U* = 2171.5, *P* = .406). Exploratory subgroup analyses showed differences in sleep latency, efficiency, and duration, but not global sleep quality, sleep disturbance, use of sleep medication, or daytime dysfunction. Black/African American participants reported greater sleep latency than White non-Hispanic and Hispanic/Latino participants (*P* = .001 for both comparisons). They also reported poorer sleep duration and sleep efficiency relative to Hispanic/Latino participants (*P* = .007 and .004, respectively).

Olfactory function differed modestly between groups. The nonminority subgroup demonstrated higher UPSIT scores (median 31 [IQR 26–32.25]) compared with the minority subgroup (median 29 [IQR 24–31]; *U* = 3365.5, *P* = .027), indicating better olfactory performance. Notably, Black/African American participants demonstrated lower UPSIT scores than all other groups; however, no pairwise comparisons remained significant in post hoc analyses.

Audiometric testing revealed significantly higher pure-tone averages (PTAs) in the nonminority subgroup across both low/mid (500, 1000, 2000 Hz) and high (2000, 4000, 8000 Hz) frequency ranges in both ears, consistent with worse hearing thresholds compared with the minority subgroup. White non-Hispanic participants demonstrated poorer hearing thresholds than Black/African American participants for left-ear low-frequency pure-tone averages (*P* = .018), right-ear high-frequency pure-tone averages (*P* < .001), and left-ear high-frequency pure-tone averages (*P* < .001). White participants also had poorer high-frequency hearing than Hispanic/Latino participants in both ears (right ear *P* = .030; left ear *P* < .001).

Cognitive performance differed between groups. Median MMSE-2:BV scores were higher in the nonminority subgroup (15.5 [IQR 15–16]) than in the minority subgroup (15 [IQR 14–15]; *U* = 3468, *P* < .001). Similarly, MMSE-2:SV scores were higher in the nonminority subgroup (29 [IQR 28–30]) compared with the minority subgroup (26 [IQR 25–28]; *U* = 3966, *P* < .001) (Table 1). Further analyses revealed that regardless of race/ethnicity, White non-Hispanic participants obtained higher MMSE-2 BV scores than both Black/African American (*P* = .041) and Hispanic/Latino participants (*P* < .001). Similarly, White participants scored higher on the MMSE-2 SV than Black/African American and Hispanic/Latino participants (both *P* < .001).

### Regression Analyses

Multiple linear regression models were constructed to evaluate predictors of MMSE-2:BV and MMSE-2:SV scores (Table 3). Stepwise selection using Akaike’s Information Criterion (AIC) favored reduced models excluding age and sleep duration for both cognitive outcomes.

For MMSE-2:BV, minority status (*β* ≈ −0.57 to −0.65, *P* < .05), GDS total score (*β* ≈ −0.08 to −0.09, *P* < .05), and UPSIT score (*β* ≈ 0.03, *P* < .05) were independently associated with cognitive performance. Minority status was associated with approximately a 0.6-point lower MMSE-2:BV score, while each additional depressive symptom corresponded to a 0.08 to 0.09-point decrease. In contrast, each one-point increase in UPSIT score was associated with a 0.03-point increase in MMSE-2:BV score. The final reduced model demonstrated modest explanatory power (adjusted *R*^2^ = 0.148; *P* < .001).

For MMSE-2:SV, minority status (*β* ≈ −1.71 to −1.73, *P* < .05), GDS total score (*β* ≈ −0.16 to −0.18, *P* < .05), and UPSIT score (*β* = 0.07, *P* < .05) remained significant predictors. Minority status was associated with approximately a 1.7-point lower MMSE-2:SV score. Each additional depressive symptom was associated with a 0.16 to 0.18-point reduction, whereas each one-point increase in UPSIT score was associated with a 0.07-point increase in MMSE-2:SV score. The reduced MMSE-2:SV model demonstrated greater explanatory strength (adjusted *R*^2^ = 0.28; *P* < .001) compared with the MMSE-2:BV model.

Age and sleep duration were not retained as significant predictors in the final models. Hearing thresholds and global sleep quality were also not significant contributors in multivariable analyses.

## Discussion

This study examined the association among sensory function, depressive symptoms, sleep quality, and cognitive performance in a diverse aging cohort recruited from both an otolaryngology clinic and an aging center. Several important findings emerged. First, minority participants demonstrated lower cognitive scores and poorer olfactory performance despite being significantly younger than the nonminority group. Second, although audiometric thresholds were worse in the nonminority subgroup, hearing measures were not independently associated with cognitive performance in multivariable models. Third, depressive symptoms and olfactory function remained significant independent predictors of cognition, whereas sleep measures and age were not retained in the final regression models. Overall, model explanatory power was modest, reflecting the multifactorial and complex nature of cognitive performance in older adults. Accordingly, these findings should be interpreted as identifying associations rather than causal relationships. Given the cross-sectional design, the observed relationships cannot establish temporal directionality, and therefore should not be used in isolation to guide clinical decision-making.

### Cognitive Performance and Minority Status

Minority status was independently associated with lower MMSE-2 scores in both brief and standard versions, even after adjustment for sensory and behavioral variables. The effect size was clinically meaningful, particularly for the MMSE-2:SV, where minority status was associated with approximately a 1.7-point lower cognitive score. These findings align with prior research demonstrating racial and ethnic disparities in cognitive test performance among minority populations.^17–21^

Importantly, the observed differences were unlikely to be explained by age alone, as minority participants were significantly younger than nonminority participants. The persistence of minority status as an independent predictor after multivariable adjustment suggests that unmeasured contextual or systemic factors may contribute to cognitive disparities. Prior studies have implicated educational quality, socioeconomic conditions, discrimination, healthcare access, and other social determinants of health.^18–21^

Taken together, these findings support a biopsychosocial model of cognitive health in which racial and ethnic disparities cannot be fully explained by chronological age or socioeconomic status alone.

### Hearing and Cognition

Contrary to prior literature,^6,22–25^ audiometric thresholds were worse in the nonminority subgroup and were not independently associated with cognitive scores in multivariable models. One explanation is the age distribution across groups, as non-minority participants were significantly older and may have had more advanced age-related hearing loss.

Additionally, pure-tone audiometry may not capture central auditory processing dysfunction or real-world communication difficulties, which may be more strongly associated with cognitive outcomes than peripheral hearing thresholds. While hearing rehabilitation remains clinically important, the present findings suggest that cognitive associations may depend on population characteristics, age structure, and the specific auditory metrics used.

### Olfaction and Cognition

Olfactory performance measured by UPSIT scores demonstrated consistent independent associations with cognitive outcomes in both regression models. Each one-point increase in UPSIT score corresponded to a measurable increase in MMSE-2 performance, with stronger effects observed in the standard version. These findings are consistent with literature suggesting that olfactory dysfunction may represent an early marker of neurodegenerative pathology and shared central nervous system involvement.

Minority participants exhibited modestly lower olfactory scores compared to nonminority participants, with Black/African American participants demonstrating lower UPSIT scores than the other groups. Given that olfactory dysfunction remained an independent predictor of cognition, smell testing may have practical clinical relevance in otolaryngology settings. However, the magnitude of association was modest, and these findings do not imply that olfactory dysfunction causes cognitive decline. Rather, olfactory dysfunction may serve as a clinical signal that additional cognitive evaluation could be warranted in selected patients.

Prior studies similarly report poorer olfactory performance among African American and Hispanic populations and demonstrate associations between olfactory dysfunction and cognitive outcomes.^26–28^ These findings support the present results and suggest that olfactory disparities cannot be fully explained by age or socioeconomic factors alone.

### Depressive Symptoms and Sleep

Depressive symptoms were independently associated with lower cognitive scores in both models. Although effect sizes per point increase were modest, the consistency of this association supports prior work identifying depression as a potential modifiable risk factor for cognitive decline.

Sleep latency differed between minority and non-minority groups; however, global sleep quality and sleep duration were not retained in final regression models. These findings suggest that while specific sleep disturbances may vary across groups, sleep quality did not independently explain cognitive performance in this cross-sectional analysis.

### Limitations

Several limitations should be considered when interpreting these findings. First, participants were not formally age-matched between groups. Notably, minority participants were significantly younger (mean age 65.37 vs 72.1 years) yet demonstrated poorer memory and sensory performance. This observation strengthens the argument that disparities are unlikely to be explained by aging alone and may reflect broader structural or biological influences. An additional limitation is that MMSE-2 BV scores were clustered near the top of the scale, raising the possibility of a ceiling effect and thereby limiting sensitivity to subtle variation in cognitive performance in this relatively high-functioning sample. Future studies may benefit from using more sensitive cognitive measures in similar populations. Second, all participants resided in Miami-Dade or Broward County, which may limit generalizability. However, similar disparities in sensory and cognitive outcomes have been reported in other U.S. populations. Third, longitudinal progression data were unavailable. The study was cross-sectional, therefore, the directionality of the observed relationships cannot be determined; poorer olfaction and higher depressive symptoms may contribute to lower cognitive performance, result from it, or co-occur because of shared underlying mechanisms. Without baseline measurements, it is not possible to determine whether participants experienced accelerated decline or entered the study with pre-existing deficits. Fourth, the study did not directly measure structural or systemic determinants of health. Prior research has demonstrated that minority populations often experience delayed diagnosis and treatment of neurocognitive disorders. African American and Hispanic patients are more likely to receive late-stage diagnoses of AD even after controlling for symptom severity and socioeconomic status.^29^ Delayed access to care may be driven by barriers such as limited availability of culturally competent providers, language differences, financial constraints, and health literacy gaps.^30^

Comorbid chronic diseases, including diabetes, cardiovascular disease, and mental health disorders are also established risk factors for cognitive decline.^31^ Inequities in preventive care utilization, sensory rehabilitation (such as hearing technology), and specialty referral patterns likely contribute to the disproportionate burden of cognitive and sensory impairment observed in minority participants.^32^ Future studies should incorporate longitudinal designs and assess contextual determinants of health, including socioeconomic status and access to care.

### Clinical Implications

The findings have several clinical implications for otolaryngology and geriatric care.

First, olfactory dysfunction and depressive symptoms may serve as practical clinical indicators associated with lower cognitive performance, even when controlling for age and hearing thresholds. However, these associations should not be interpreted as causal and are not sufficient, on their own, to direct patient management. Rather, incorporating brief smell testing and depression screening into the evaluation of older adults presenting with sensory complaints may help identify patients who could benefit from more comprehensive cognitive assessment. Second, the lower cognitive and olfactory performance among minority participants despite younger age underscores the importance of equitable access to screening, culturally responsive counseling, and timely referral pathways. Finally, these findings should be viewed as hypothesis-generating and supportive of a broader risk awareness, rather than evidence of specific intervention strategies.

## Conclusion

In this diverse aging cohort, minority status, depressive symptoms, and olfactory dysfunction were independently associated with lower cognitive performance, whereas hearing thresholds and global sleep quality were not retained in multivariable models. These findings highlight the multifactorial and modest nature of sensory–cognitive associations and support the potential clinical value of incorporating olfactory and mood assessments into otolaryngologic practice. Future longitudinal research should explore causal mechanisms and examine structural, environmental, and biological pathways contributing to disparities in sensory and cognitive aging.

## Figures and Tables

**Table 1.** Sensory Survey Results by Minority Status (n = 164)

Variable	Status				*P*-value
	Minority		Nonminority		
	Mean (SD)	Median [IQR]	Mean (SD)	Median [IQR]	
Age (years)	65.37 (8.09)	65 [59–71]	72.1 (9.46)	75 [67.5–79]	<.001*
GDS Total Score (n = 112)	2.66 (2.76)	2 [1–3]	1.29 (2.04)	1 [0–2]	<.001*
Sleep Scores					
Component 1: Subjective Sleep Quality	1.02 (0.937)	1 [0–1]	0.96 (0.680)	1 [0.75–1]	.911
Component 2: Sleep Latency	1.50 (1.06)	2 [1, 2]	0.69 (0.90)	0 [0–1]	<.001*
Component 3: Sleep Duration	1.17 (0.93)	1 [1, 2]	1.07 (0.86)	1 [0–2]	.609
Component 4: Sleep Efficiency	1.36 (1.34)	1 [0–3]	1.4 (1.34)	1 [0–3]	.948
Component 5: Sleep Disturbance	1.37 (0.72)	1 [1, 2]	1.29 (0.59)	1 [1, 2]	.5
Component 6: Use of Sleep Medication	0.79 (1.22)	0 [0–2]	0.80 (1.25)	0 [0–1]	.856
Component 7: Daytime Dysfunction	0.60 (0.72)	0 [0–1]	0.6 (0.65)	1 [0–1]	.805
Global PSQI Score:	7.77 (4.67)	7 [4–11]	6.82 (3.50)	7 [4–9]	.406
UPSIT Score	26.7 (6.63)	29 [24–31]	28.75 (6.60)	31 [26–32.25]	.027*
Hearing					
PTA 500, 1 KHz, 2 KHz right ear	26.51 (18.96)	20 [15–31.25]	31.67 (17.86)	25 [18.33–41.67]	.020*
PTA 500, 1 KHz, 2 KHz left ear	26.04 (16.55)	21.67 [15–30]	32.86 (20.05)	27.5 [22.08–39.58]	.006*
PTA 2 KHz, 4 KHz, and 8 KHz right ear	35.53 (22.02)	31.67 [20–45]	48.59 (22.11)	33.33 [15–48.33]	<.001*
PTA 2 KHz, 4 KHz, and 8 KHz left ear	34.44 (22.02)	29.17 [20–45]	52.39 (23.48)	48.33 [40–61.25]	<.001*
MMSE-2:BV Total Raw Score	14.47 (1.13)	15 [14, 15]	15.24 (0.923)	15.50 [15, 16]	<.001*
MMSE-2:SV Total Raw Score	26.57 (2.01)	26 [25–28]	28.72 (1.44)	29 [28–30]	<.001*

* *P*-value is determined to be significant at the .05 level; *t*-tests or Mann-Whitney *U* tests were used, where appropriate.

**Table 2.** Sensory Results by Race & Ethnicity (n = 164)

	Race								
Variable	Black/African American, non-Hispanic (n = 49) Mean (SD)	Median [IQR]	Hispanic/Latino (n = 62) Mean (SD)	Median [IQR]	White, non-Hispanic (n = 48) Mean (SD)	Median [IQR]	Other, non-Hispanic (n = 4) Mean (SD)	Median [IQR]	*P*-value
Age (years)	62.92 (6.07)	62 [59–67]	67.06 (8.93)	67 [60–74.75]	72.1 (9.46)	75 [67.5–79]	69.25 (9.95)	69 [63.25–75]	<.001*
GDS Total Score (n = 112)	3.11 (3.09)	2 [1–5]	2.36 (2.51)	2 [1–3]	1.29 (2.04)	1 [0–2]	2.25 (2.22)	2 [0.75–3.5]	.−002*
Sleep Scores Component	1.19 (0.98)	1 [0.5–2]	0.92 (0.92)	1 [0–1]	0.96 (0.68)	1 [0.75–1]	0.75 (0.50)	1 [0.75–1]	.476
1: Subjective Sleep Quality									
Component 2: Sleep Latency	1.58 (1.10)	2 [1–2.5]	1.47 (1.04)	1 [1, 2]	0.69 (0.90)	0 [0–1]	1 (1.16)	1 [0–2]	<.001*
Component 3: Sleep Duration	1.54 (0.96)	1 [1, 2]	0.92 (0.84)	1 [0–1]	1.07 (0.86)	1 [0–2]	1 (0.82)	1 [0.75–1.25]	.011*
Component 4: Sleep Efficiency	1.93 (1.35)	3 [0–3]	1 (1.19)	0.5 [0–2]	1.4 (1.34)	1 [0–3]	0.75 (1.50)	0 [0–0.75]	.005*
Component 5: Sleep Disturbance	1.35 (0.84)	1 [1, 2]	1.40 (0.61)	1 [1, 2]	1.29 (0.59)	1 [1, 2]	1 (0.82)	1 [0.75–1.25]	.632
Component 6: Use of Sleep Medication	1.02 (1.35)	0 [0–3]	0.66 (1.14)	0 [0–1]	0.80 (1.25)	0 [0–1]	0.25 (0.50)	0 [0–0.25]	.498
Component 7: Daytime Dysfunction	0.63 (0.76)	0 [0–1]	0.60 (0.71)	0 [0–1]	0.60 (0.65)	1 [0–1]	0.25 (0.50)	0 [0–0.25]	.79
Global PSQI Score:	9.23 (4.97)	9 [5–12.5]	6.20 (4.30)	7 [4–10]	6.82 (3.50)	7 [4–9]	5 (2.71)	6 [4.75–6.25]	.057
UPSIT Score	24.92 (8.20)	27 [19–31]	28.29 (4.58)	29 [26.25–31]	28.75 (6.60)	31 [26–32.25]	24 (7.44)	27 [22.75–28.25	.032*
Hearing PTA 500, 1 KHZ, 2 KHZ, right ear	24.83 (18.91)	20 [16.25–30]	25.55 (15.38)	21.67 [15–31.67]	31.67 (17.86)	25 [18.33–41.67]	42.08 (29.10)	46.67 [24.17–64.58]	.047*
PTA 500, 1 KHZ, 2 KHZ, left ear	23.96 (13.91)	21.67 [15–25.42]	25.74 (15.97)	21.67 [15–30]	32.86 (20.05)	27.5 [22.08–39.58]	49.58 (34.33)	50 [32.08–67.50]	.011*
PTA 2 KHZ, 4KHz, and 8 KHz, right ear	30.69 (21.10)	25.83 [16.67–36.67]	36.91 (19.18)	35 [20–48.33]	48.59 (22.11)	45.83 [33.33–64.58]	51.25 (28.56)	57.5 [41.67–67.08]	<.001*
PTA 2 KHZ, 4KHz, and 8 KHz, Left ear	29.13 (20.16)	23.33 [16.67–37.50]	36.48 (20.92)	33.33 [21.67–46.67]	52.39 (23.48)	48.33 [40–61.25]	60.83 (38.26)	65 [42.92–82.92]	<.001*
MMSE-2 BV Total Raw Score	14.64 (1.13)	15 [14–15.5]	14.37 (1.13)	15 [14, 15]	15.24 (0.92)	15.5 [15, 16]	14 (0.82)	14 [13.75–14.25]	<.001*
SV Total Raw Score	26.65 (2.04)	26 [25–28]	26.54 (2.01)	27 [25–28]	28.72 (1.44)	29 [28–30]	26 (2.16)	26.5 [25.25–27.25]	<.001*

Values are presented as mean (SD) and median [IQR].

* *P* < .05. When the omnibus test was significant, post hoc pairwise comparisons were conducted using Wilcoxon rank-sum tests with adjustment for multiple comparisons.

**Table 3.** Regression Modeling Coefficients Summary

Test	Minority status	Age (years)	GDS Total Score	Sleep duration	UPSIT Score	AIC	Adj *R*^2^	*P*-value
MMSE-2:BV	−0.65*	−0.012	−0.091*	0.12	0.03*	423.722	0.15	<.001*
MMSE-2:BV	−0.571*	-	−0.083*	0.145	0.033*	423.188	0.148	<.001*
MMSE-2:SV	−1.710*	0.001	−0.178*	0.168	0.070*	586.102	0.275	<.001*
MMSE-2:SV	−1.729*	-	−0.164*	-	0.070*	583.084	0.28	<.001*

* Denotes statistical significance at the 0.05 level.
